# Cerebrospinal fluid fistulas after iliosacral screw removal in post-traumatic pseudomeningocele

**DOI:** 10.1007/s10195-011-0163-x

**Published:** 2011-11-03

**Authors:** Angiola Valente, Alberto Nicodemo, Antonio Bruno, Alessandro Massè

**Affiliations:** 1 Department of Orthopaedic and Traumatology, San Luigi Gonzaga Hospital, University of Turin, Regione Gonzole n. 10, 10043 Orbassano, TO Italy; 2 CTO/Maria Adelaide Hospital, Turin, Italy

**Keywords:** Pseudomeningocele, Cerebrospinal fluid fistulas, Sacral fracture, Complication

## Abstract

Sacral fractures are rare but severe injuries. They are often associated with neurological impairment and pelvic instability. We present a case of a 28-year-old woman who sustained an H-type fracture of the sacrum with complete cauda equina syndrome treated with cauda equina decompression and pelvic percutaneous stabilization with an iliosacral screw. Two years after she underwent screw removal, but complained of back and nape pain after the operation. A lumbosacral MRI showed the presence of a lytic lesion involving the S1 and S2 bodies that was judged to be a pseudomeningocele leaning against the sacral screw hole and cerebrospinal fluid fistulas through this. To our knowledge, this is the first case of such a complication after sacral screw removal to be reported.

## Introduction

The incidence of pelvic fractures is about 30 per 100,000/year. Involvement of the sacrum occurs in approximately 45% of the cases [1], and is more frequent in cases of high-energy trauma.

Numerous techniques have been advocated for the fixation of posterior pelvic ring and sacral fractures [2, 3]; among these, the use of iliosacral or transiliosacral screws is widely accepted to be the gold standard due to its mechanical stability [4]. Various complications of this technique have been described in the literature: nerve injury, misplaced screws, fixation failures, infection, and lack of posterior pelvic reduction [4, 5].

In this report, we describe a case of symptomatic cerebrospinal fluid fistulas after sacral screw removal in a young woman. To our knowledge, this complication has never been described before.

## Case report

**Fig. 1:**
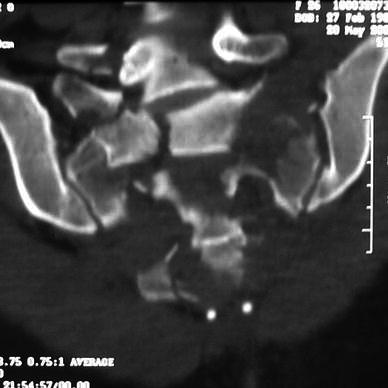
Axial CT-scan image illustrating the H-type fracture of the sacrum

A 28 year-old healthy woman was involved in a motorbike accident in May 2007. She sustained a right T-type acetabular fracture and an AO/OTA 61 C1-3 type pelvic fracture (Fig. 1) consisting of an H-type fracture of the sacrum and of the pubic and ischial rami. Neurological examination at admission showed complete cauda equina syndrome (with urinary retention, sexual dysfunction, and saddle anesthesia).

**Fig. 2:**
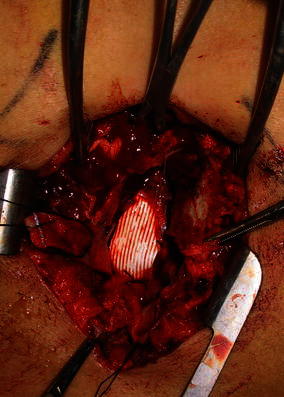
Application of the Gore-Tex sacral patch

Due to the hemodynamic instability and the neurological damage, the patient underwent urgent cauda equina decompression with Gore-Tex patch application (Fig. 2), pelvic percutaneous stabilization under fluoroscopy with an 8 mm stainless steel transiliosacral screw [1] (Asnis, Stryker, Howmedica, Mahwah, NJ, USA) and external fixation of the pelvis (BlueShark, Mikai, Genova, Italy). The patient was dismissed from the intensive care unit after five days.

Eight days after the trauma, open reduction and internal fixation of the T-shaped acetabular fracture was performed by a modified Kocher–Langenbeck approach with surgical dislocation of the hip according to Ganz [3].

**Fig. 3:**
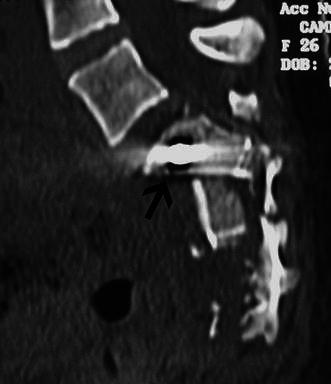
Sagittal CT image illustrating the correct position of the sacral screw immediately after the operation (*arrow*)

A postoperative CT of the pelvis showed the correct placement of the iliosacral screw inside the body of S1 (Fig. 3).

The patient was discharged from the hospital 15 days after without any further complications. Three months after surgery she was walking without pain and she had a good range of movement of the right hip. The lesion of the cauda equina did not show any recovery.

She was admitted to our hospital in June 2009 to remove implants under general anesthesia because of the local pain caused by the heads of the trochanteric screws and the trans-sacral screw. She was discharged the day after the procedure with partial weight-bearing indicated for two weeks. Two days after, she went to the emergency department complaining of intense back and nape pain associated with difficulty walking and standing. The pain was reduced in the supine position.

**Fig. 4:**
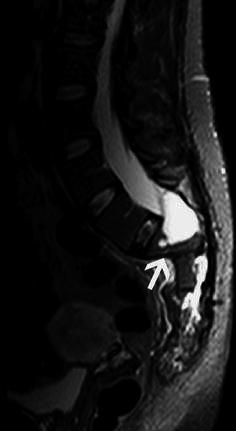
Sagittal T1/T2-weighted MR image illustrating anterior pseudomeningocele (*arrow*)

**Fig. 5:**
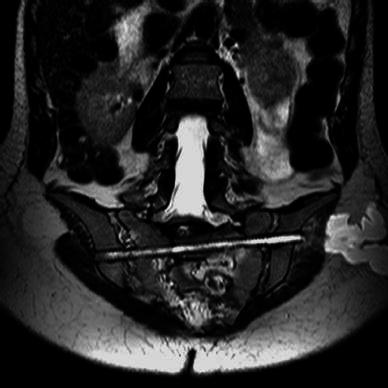
Axial T1/T2-weighted MR image illustrating CSF runoff through the screw hole after removal

A lumbosacral MRI was performed showing the presence of a lytic lesion involving the S1 and S2 bodies, which was judged to be a pseudomeningocele leaning against the sacral screw hole (Figs. 4, 5).

The neurological diagnosis was liquor hypotension due to the interruption of the pseudomenincocele wall by the screw thread during its removal. The patient was evaluated by a neurosurgeon, who decided not to operate; just follow her clinically and radiologically. The therapy was bed rest and hydration. Nine days after she showed complete remission of the symptoms. At six months follow-up, the patient was healthy, even though there was no improvement in the neurological symptoms.

The patient gave her informed consent prior to being included in the study.

## Discussion

Seventy-eight sacral fractures were treated at our center from 2002 to September 2010 with 116 trans-sacral or iliosacral screws. The complications of this cohort were a lack of reduction (eight cases), one trans-sacral screw rupture, and one sacral nonunion.

Anterior pseudomeningocele after trauma is a rare entity [6]. Pseudomeningocele is an extradural collection of cerebrospinal fluid (CSF) that results from a dural breach [7]. The causes may be classified into three categories: iatrogenic (the most common), traumatic, and congenital. The incidence of iatrogenic pseudomeningocele ranges from 0.3 to 13%. Trauma-related pseudomeningoceles are rare and the true incidence is unknown [8]. Traumatic pseudomeningoceles are usually associated with a nerve root stretch injury with possible avulsion of the nerve root itself [9, 10]. The dural tear is walled off by proliferation of dura and arachnoid mater, and the cyst enlarges because of a pressure differential [9].

They are even more rare at the sacral level than those higher on the spine because of the stability of the bony pelvis [9, 10]. For the case described here, a possible explanation of the anterior development of the pseudomeningocele with sacral erosion could be a complete and extensile lesion of the dura at the fracture level and the posterior closure of the lesion with a synthetic patch; as the pseudomeningocele was lying against the smooth shaft of the screw, its wall was damaged by the screw thread during its removal, thus causing liquorrhea.

MRI was not performed after her first hospital admission. After a sacral fracture associated with cauda equina syndrome, a follow-up MRI may be useful for diagnosing a post-traumatic pseudomeningocele, thus avoiding this kind of complication.
